# Mechanical evidence that *Australopithecus sediba* was limited in its ability to eat hard foods

**DOI:** 10.1038/ncomms10596

**Published:** 2016-02-08

**Authors:** Justin A. Ledogar, Amanda L. Smith, Stefano Benazzi, Gerhard W. Weber, Mark A. Spencer, Keely B. Carlson, Kieran P. McNulty, Paul C. Dechow, Ian R. Grosse, Callum F. Ross, Brian G. Richmond, Barth W. Wright, Qian Wang, Craig Byron, Kristian J. Carlson, Darryl J. de Ruiter, Lee R. Berger, Kelli Tamvada, Leslie C. Pryor, Michael A. Berthaume, David S. Strait

**Affiliations:** 1 Department of Anthropology, University at Albany, 1400 Washington Avenue, Albany, New York 12222, USA; 2 Zoology Division, School of Environmental and Rural Science, University of New England, Armidale, New South Wales 2351, Australia; 3 Department of Anthropology, Washington University in St. Louis, St. Louis, Missouri 63130, USA; 4 Department of Cultural Heritage, University of Bologna, Via degli Ariani 1, Ravenna 48121, Italy; 5 Department of Human Evolution, Max Planck Institute for Evolutionary Anthropology, Deutscher Platz 6, 04103 Leipzig, Germany; 6 Department of Anthropology, University of Vienna, Althanstrasse 14, A-1090 Vienna, Austria; 7 Department of Biology, South Mountain Community College, 7050 South 24th Street, Phoenix, Arizona 85042, USA; 8 Department of Anthropology, Texas A&M University, College Station, Texas, 77843, USA; 9 Department of Anthropology, University of Minnesota, 395 Hubert H. Humphrey Center, 301 19th Avenue South, Minneapolis, Minnesota 55421, USA; 10 Department of Biomedical Sciences, Texas A&M University Baylor College of Dentistry, 3302 Gaston Avenue, Dallas, Texas 75246, USA; 11 Department of Mechanical & Industrial Engineering, University of Massachusetts, 160 Governor's Drive, Amherst, Massachusetts 01003-2210, USA; 12 Department of Organismal Biology & Anatomy, University of Chicago, 1027 East 57th Street, Chicago, Illinois 60637, USA; 13 Division of Anthropology, American Museum of Natural History, New York, New York 10024, USA; 14 Department of Anatomy, Kansas City University of Medicine and Biosciences, 1750 Independence Avenue, Kansas City, Missouri 64106-1453, USA; 15 Department of Biology, Mercer University, 1400 Coleman Avenue, Macon, Georgia 31207, USA; 16 Evolutionary Studies Institute, University of the Witwatersrand, Palaeosciences Centre, Private Bag 3, Wits 2050, South Africa; 17 Department of Anthropology, Indiana University, Bloomington, Indiana 47405, USA; 18 Department of Biology and Health Sciences, The Sage Colleges, 65 First Street, Troy, New York 12108, USA; 19 Max Planck Weizmann Center for Integrative Archaeology and Anthropology, Max Planck Institute for Evolutionary Anthropology, Deutscher Platz 6, Leipzig 04103, Germany

## Abstract

*Australopithecus sediba* has been hypothesized to be a close relative of the genus *Homo.* Here we show that MH1, the type specimen of *A. sediba*, was not optimized to produce high molar bite force and appears to have been limited in its ability to consume foods that were mechanically challenging to eat. Dental microwear data have previously been interpreted as indicating that *A. sediba* consumed hard foods, so our findings illustrate that mechanical data are essential if one aims to reconstruct a relatively complete picture of feeding adaptations in extinct hominins. An implication of our study is that the key to understanding the origin of *Homo* lies in understanding how environmental changes disrupted gracile australopith niches. Resulting selection pressures led to changes in diet and dietary adaption that set the stage for the emergence of our genus.

## Abstract (web summary)

Dietary adaptations of extinct early humans are often inferred from dental microwear data. Here, the authors employ mechanical analyses to show that *Australopithecus sediba* had limited ability to consume hard foods.

## Graphical abstract

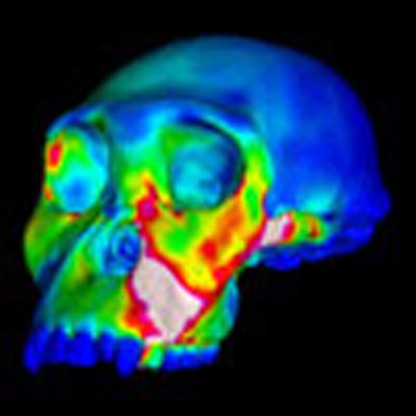

Recent years have seen a number of increasingly sophisticated studies^1^,^2^,^3^,^4^,^5^,^6^,^7^ directed towards reconstructing the diets of australopiths (extinct early humans from the Plio-Pleistocene of Africa). These studies suggest that australopiths exhibit dietary diversity both within and between species, and challenge conventional wisdom concerning australopith dietary adaptations that have traditionally been based on assessments of functional anatomy^8^,^9^. Consequently, a lively debate has ensued concerning the efficacy of functional studies^10^,^11^,^12^. We demonstrate here that mechanical evidence provides insights into the dietary adaptations of *Australopithecus sediba* that could not have been obtained from dietary reconstructions that do not consider functional anatomy. These insights may elucidate some of the selective factors that led to the origin of our genus. Indeed, even though the precise phylogenetic relationships of *A. sediba* are a matter of debate (competing hypotheses place it as either the sister taxon of *Homo*^13^ or a phyletic descendant of *A. africanus* lacking a close relationship with early *Homo* taxa^14^), we show that the adaptive implications of its feeding mechanics are the same in all relevant phylogenetic scenarios.

*Australopithecus sediba*, known from Malapa in southern Africa^13^, is unlike other australopiths in that it has comparatively small molar and premolar teeth, lacks large muscle markings, and exhibits only a few traits that might increase muscle leverage and/or buttress the face^13^,^15^. Yet, despite its apparent gracility, dental microwear analysis suggests that its diet included hard foods^7^. Such foods presumably would be fractured on the molars and premolars using high bite forces (if they were instead fractured using stone tools, then microwear would not detect evidence of hard-object feeding). Thus, dietary evidence seems to be at odds with conventional interpretations of functional anatomy. We test the hypothesis that the facial skeleton of *A. sediba* is well configured (and, possibly, adapted) to consume hard foods. This hypothesis predicts that the cranium of *A. sediba* is structurally strong in response to feeding loads, and that it is able to efficiently generate high bite forces on its molars and premolars.

We tested hypotheses about feeding biomechanics using finite element analysis (FEA), an engineering technique useful for examining the specific functional consequences of cranial shape variation^16^. A finite element model (FEM) of the *A. sediba* holotype cranium (MH1) was virtually reconstructed to correct for damaged, displaced or missing parts. This model was compared with a FEM of *A. africanus*, a composite model of Sts 5, and Sts 52a and b^10^,^17^. Sts 5 was also used to reconstruct the missing occipital region of MH1, so both models are composites. However, occipital morphology does not figure in the mechanical analyses reported here. For simplicity, the *A. africanus* and *A. sediba* FEMs are referred to here as the specimens on which they are primarily based, namely Sts 5 and MH1, respectively. Both models were subjected to loads simulating maximal bites on the left upper third premolar (P^3^) and left upper second molar (M^2^), under the assumption that the chewing muscles were acting at peak activity levels on both sides of the cranium. These loads allow an estimate of the maximum bite force produced by each individual. We have previously shown that FEA of primate crania can produce maximum bite force calculations within 5% of values recorded during *in vivo* experiments^10^. Muscle force magnitudes were scaled such that bone strain differences in the two models reflect differences in cranial shape but not size^18^. FEA simulates rather than directly measures the forces, stresses and strains associated with behaviours performed by living organisms, so results from FEA are approximate. However, a great analytical strength of FEA is that it allows researchers to test questions under absolutely controlled conditions at a level of complexity beyond what can generally be achieved in a laboratory setting, especially when examining extinct taxa known only from fossils. We use FEA to show that the cranium of *A. sediba* is not optimized to produce high molar bite forces and therefore is unlikely to have been adapted to eat hard foods.

## Results

### Strain and bite force

**Figure 1:**
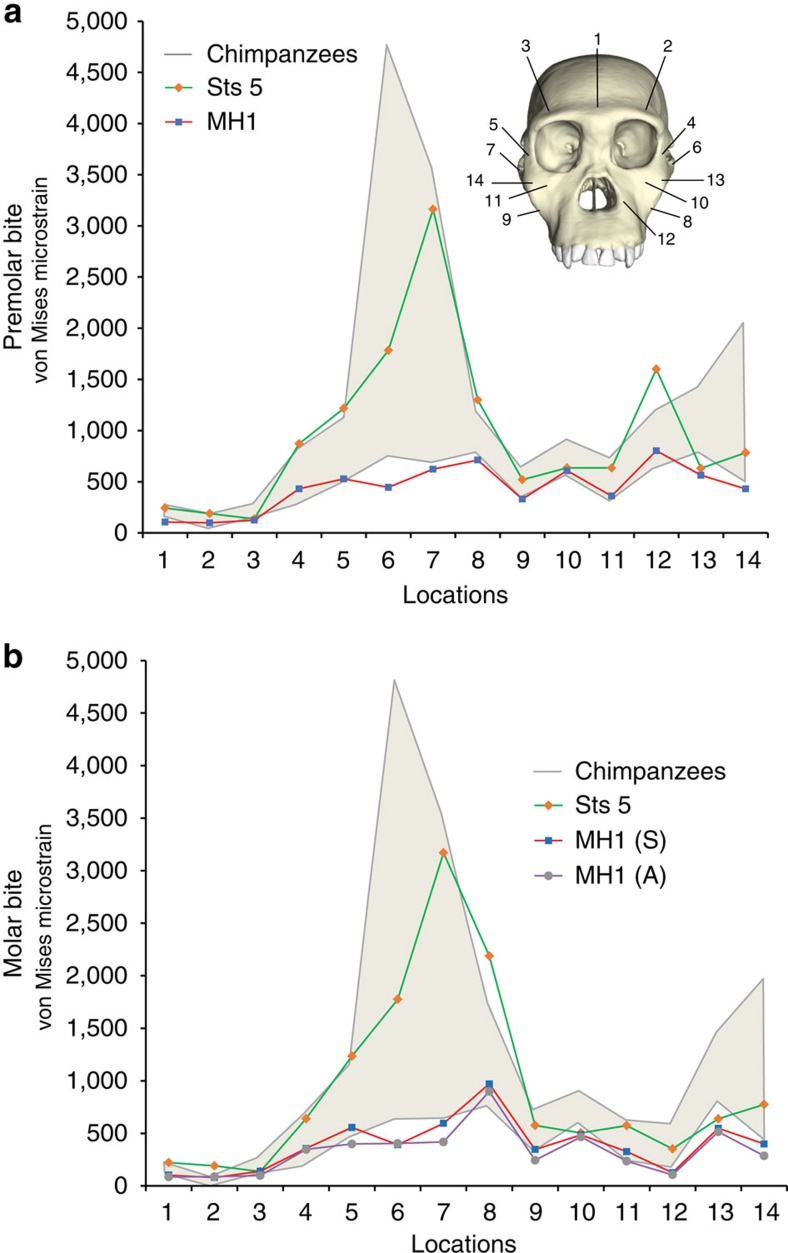
Line plot of von Mises strain generated during simulated biting in finite element models. Strain data correspond to (**a**) left premolar (P^3^) and (**b**) left molar biting (M^2^), recorded from 14 homologous locations across the craniofacial skeleton of finite element models of Sts 5 (*A. africanus*) and MH1 (*A. sediba*). The grey region brackets the range of variation exhibited by six chimpanzee crania intentionally selected to be morphologically different. For the molar biting analysis, it was necessary to rerun the model of MH1 with the balancing (non-biting) side muscle forces reduced by nearly 30% to remove a distractive (tensile) reaction force at the working (biting) side jaw joint. Therefore, data for both the symmetrical (S) and asymmetrical (A) loadings are shown.

**Figure 2:**
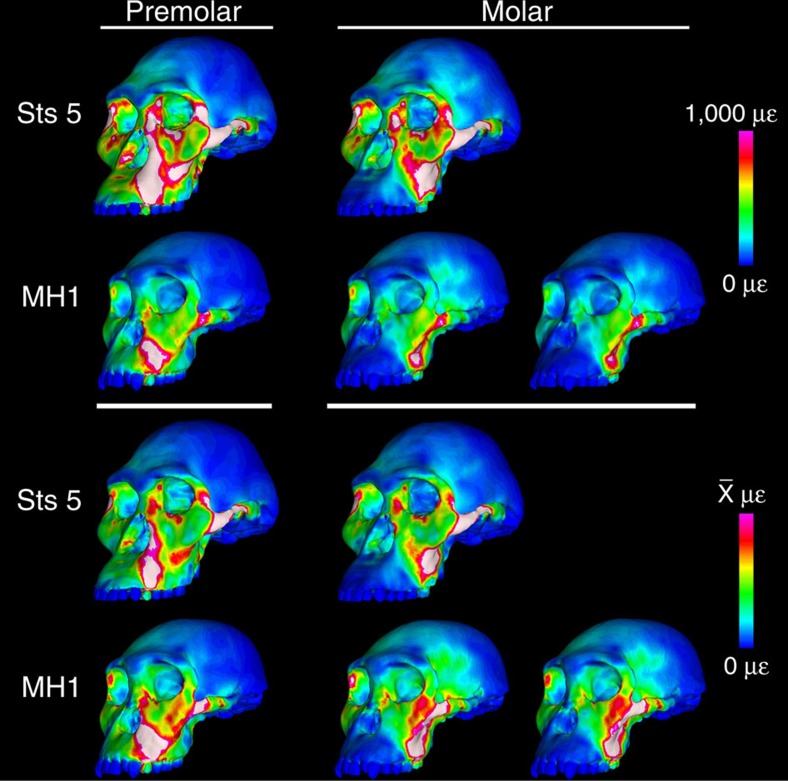
Colour mapping of von Mises strain in finite element models of Sts 5 (*A. africanus*) and MH1 (*A. sediba*) crania during simulated left premolar (P^3^) and left molar (M^2^) biting (not to scale). Colour maps on the top half of the figure reflect absolute strain magnitudes ranging from 0 to 1,000 microstrain (μɛ), where white regions experience strain magnitudes that exceed 1,000 μɛ. Colour maps on the bottom half of the figures reflect relative strain magnitudes where the colour scale of each model ranges from 0 to a value equal to twice the average of strain magnitudes collected from 10 standard locations from which strain data have been collected during *in vivo* feeding experiments in primates^29^. These relative strain maps provide information about the distribution of relatively high and relatively low strain concentrations independent of the scale of the strain magnitudes. For the molar biting analysis, it was necessary to rerun the model of MH1 with the balancing (non-biting) side muscle forces reduced by nearly 30% in order to remove a distractive (tensile) reaction force at the working (biting) side jaw joint. Therefore, under the column for molar biting, the model of MH1 is shown loaded with bilaterally symmetrical muscle forces (left), as well as with asymmetrical muscle forces (right).

Our results show that the facial skeleton of *A. sediba* specimen MH1 was strong relative to that of *A. africanus* specimen Sts 5 even though the former lacks many of the derived facial buttressing features seen in other australopiths. With a few exceptions, strain magnitudes from homologous locations on the FEMs were lower in the *A. sediba* model than in the *A. africanus* model during both premolar and molar biting (Figs 1 and 2). Nearly, all regions we examined experienced lower strain magnitudes in *A. sediba* for all forms of strain during both premolar (Supplementary Table 1) and molar (Supplementary Table 2) biting. The only exceptions to this are that *A. sediba* exhibited slightly higher maximum principal (tensile) strain in the balancing-side zygomatic root during both premolar and molar biting. The magnitude of minimum principal (compressive) strain was greater in the balancing dorsal orbital, working infraorbital and working zygomatic body regions of MH1, for both bites. There was also somewhat higher maximum shear strain in the working infraorbital during premolar biting, and somewhat higher shear and von Mises strain in the balancing dorsal orbital of MH1. However, for the infraorbital region, *A. africanus* exhibits a region of high strain just medial to where the working infraorbital strain was collected that extends from the inferior orbital rim to the root of the zygomatic. This is likely related to the more curved zygomaticoalveolar crest of Sts 5 combined with its more laterally flaring zygoma. Strain energy density in the balancing dorsal orbitals of *A. sediba* and *A. africanus* were identical. Strain mode also differed slightly between the models. Strain mode for the working postorbital bar was somewhat more compressive in *A. sediba* during premolar biting, as opposed to being tensile in *A. africanus*. In addition, the balancing mid-zygomatic arch of *A. sediba* was tensed slightly more than it was compressed during both premolar and molar biting, while compression was dominant in the arch of *A. africanus*.

Although Sts 5 experiences systematically higher strain magnitudes than MH1, the extent of the differences between these two hominins does not exceed that observed among a sample of chimpanzees that differ notably in shape (Fig. 1); strains in Sts 5 are often towards the high end or middle of the chimpanzee range, while those in MH1 are consistently toward the bottom of or below that range. Thus, although one can conclude that MH1 is strong relative to the other crania, more work is needed to determine whether or not that strength reflects a species-level property differentiating *A. sediba* from chimpanzees and *A. africanus.*

Moreover, the spatial patterning of strains in MH1 and Sts 5 is similar. It is possible to visualize the spatial patterning of strains by altering the scales of the colour maps such that they range from zero to a value equal to twice the average of 10 regions from which strain data were collected (Supplementary Tables 1 and 2, regions 1–5 and 8–12; regions 6 and 7 are excluded because strain values in those regions are highly variable). The most notable difference is that Sts 5 lacks well-defined strain concentrations in the body of the zygomatic, which exhibits a distinct prominence. The similarities in strain distribution suggest that although the hominins considered here belong to different species, they appear to share at least aspects of an underlying architecture that results in their crania performing biomechanically in similar ways despite evident variation in morphology, particularly with respect to facial projection and the shape and relative position of the zygomatic. As defined using a conventional taxonomy, *A. africanus* exhibits variation in facial projection and zygomatic root morphology (for example, specimens Sts 5 versus Sts 71), and we hypothesize that the modelling of additional individuals in this species will reveal corresponding variation in facial strain magnitudes (with some individuals possibly resembling *A. sediba*) but broad similarities in strain patterning. Such work should also establish whether or not the distribution of strain concentrations in MH1 differs from those of the other *A. africanus* specimens in the same, subtle way that it differs from Sts 5.

During simulated maximal bites with bilaterally symmetrical external forces representing fully active muscles, *A. sediba* exhibited bite forces broadly similar to those of *A. africanus* at the P^3^ and M^2^ (Table 1). The efficiency of bite force production, as measured by mechanical advantage (the ratio of bite force output to muscle force input) was slightly lower in *A. sediba* during premolar biting, but higher during molar biting compared with *A. africanus*.

**Figure 3:**
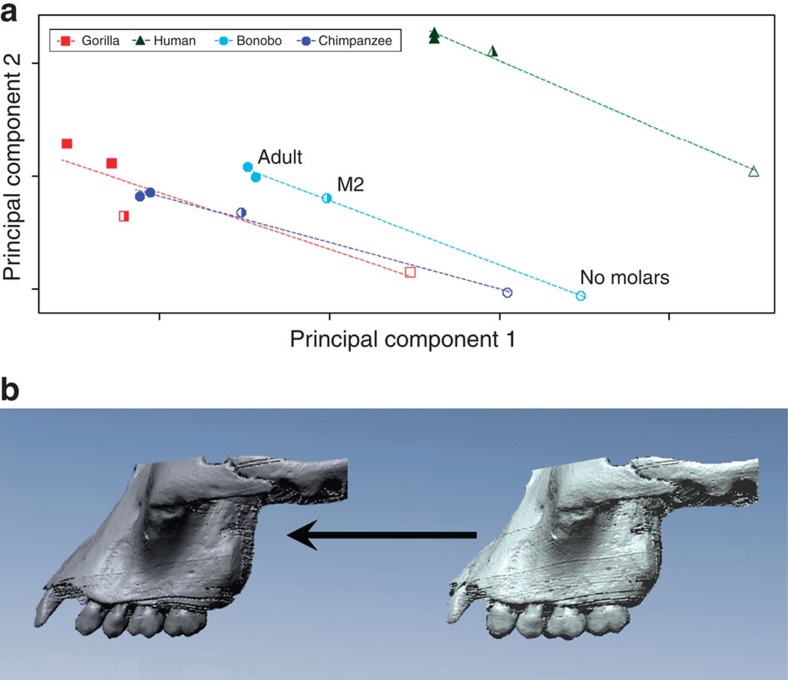
Ontogenetic changes among the dentition, malar root and temporomandibular joint in 319 extant African apes and humans. (**a**) Principal component summary of shape differences, represented by mean configurations, among specimens with no molars (open symbols), M2 in occlusion (partially filled symbols) and adults (filled symbols). (**b**) Surface reconstruction of MH1 specimen rendered from down-sampled synchotron scans (right) and hypothetical adult morphology of MH1 generated using a male chimpanzee developmental trajectory (left).

These results have not been biased by the subadult age of MH1. Previous studies have demonstrated in apes^19^, monkeys^20^ and other hominins^19^,^21^,^22^,^23^,^24^ that developmental changes in overall cranial morphology that occur subsequent to first molar eruption produce relatively minor variations in shape relative to the changes that occur earlier in ontogeny. Moreover, one study in Old World monkeys has shown that many (although not all) components of the masticatory system exhibit consistent spatial relationships with each other during growth from juvenile stages to adulthood^25^. Our geometric morphometric analysis of ontogenetic change in African apes and humans suggests that the important functional relationships in MH1 among the dentition, malar root and temporomandibular joint (TMJ) would likewise have not changed markedly during the later stages of development. These are key landmarks to consider because collectively they affect the relative positioning of critical components of the biomechanical system, namely, the point of loading (that is, the bite point), some of the applied external forces (that is, the masseter muscle) and the fulcrum (TMJ). If, for example, the positions of the tooth row relative to the zygomatic root and TMJ were markedly different in adults compared with juveniles of the age of MH1, then results derived from MH1 might have limited utility in characterizing the mechanics of the species. However, relative to African ape juveniles lacking permanent teeth, those individuals with the second molar in occlusion have nearly achieved the adult configuration in these features (Fig. 3a). And, while subsequent ontogenetic changes are measurable, they do not substantially alter the arrangement of the masticatory apparatus (Fig. 3b). In other words, even if MH1 had lived to grow along the ontogenetic trajectory of an extant ape, the resulting adult form would have been very similar to that preserved in the existing fossil with respect to the morphological features under consideration. Moreover, even though *A. sediba* is represented in this study by a single individual, prior work on chimpanzee crania has shown that mechanical variation within species is conservative even when intraspecific shape variation is high^26^. Thus, even though it is always desirable to examine more specimens (particularly in fossil taxa), there is reason to believe that these results are broadly applicable to the species as a whole, with the caveat that mechanical variation at approximately the level seen in chimpanzees might be expected.

### Constraints

**Figure 4:**
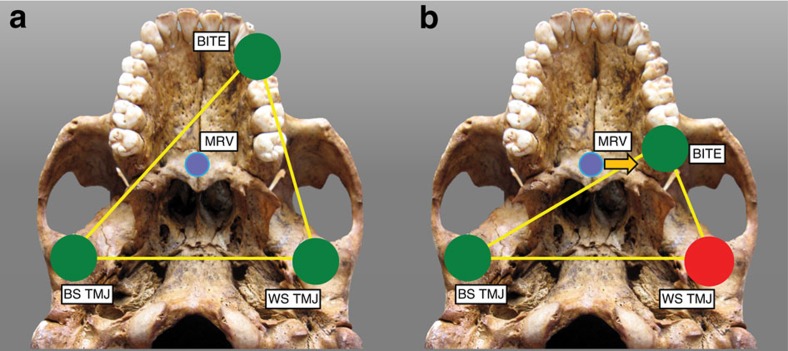
The constrained lever model of jaw biomechanics. A ‘triangle of support' is formed by the bite point (BITE) and the working-side (WS) and balancing-side (BS) temporomandibular joints (TMJ). During a premolar bite (**a**), the muscle resultant vector (MRV) of the jaw adductor (masticatory) muscles remains within the triangle (passing into the plane of the image), producing compression (green circles) at all three points as the mandible is elevated. However, during some molar bites (**b**), the MRV falls outside the triangle when the muscles are being recruited equally on both sides of the head, producing compression at the bite point and BS TMJ, but distraction (red circle) at the WS TMJ. To eliminate the distraction, the recruitment of the balancing-side muscles must be lessened, thereby causing the MRV to shift its position towards the working side (arrow). Once the MRV falls back within the triangle, then the WS TMJ will be in compression. A consequence of reducing the recruitment of the balancing-side muscles is that the magnitude of the bite force is reduced.

On balance, one might conclude that the cranium of MH1 is well configured to consume hard foods, insofar as its facial skeleton is structurally strong (notwithstanding sampling limitations) and it appears to produce bite force efficiently. However, there is an important constraint on bite force production that argues against this interpretation. In this ‘constrained lever' model^27^,^28^ (Fig. 4), it is assumed that the two TMJs are loaded in compression (in which the mandibular condyles are drawn into the joints). This will occur when the vector resultant of all of the masticatory muscle forces passes through a ‘triangle of support' defined by the bite point and the two TMJs. If, however, the muscle resultant were to fall outside of the triangle of support, then the working (biting) side TMJ would experience a distractive reaction force in which the condyle is ‘pulled' out of the joint. The soft tissues of the TMJ are poorly configured to resist distractive joint forces, and thus could be damaged if the joint were to dislocate in this manner.

The muscle resultant will fall in the midline (that is, the midsagittal plane) when the muscles of mastication are acting with bilateral symmetry (equal forces on both sides)^27^,^28^. As a generalization, such a midline muscle resultant passes through the triangle of support during bites on the mesial teeth (that is, incisors, canines and premolars). Thus, the model^27^ does not predict distraction during bites on such teeth. However, as the bite point moves distally on the tooth row towards the molars, the shape of the triangle of support changes such that a midline muscle resultant may lie outside of the triangle (Fig. 4), and thus create a distractive joint force. To bring the resultant within the triangle, one can reduce the activity levels of the balancing (non-biting) side muscles^27^,^28^. This has the effect of moving the muscle resultant towards the working side and back within the triangle (Fig. 4). In such bites, there is an asymmetry in the activity levels of the working and balancing-side muscles, a consequence of which is that overall muscle force is reduced. Thus, although one might expect that a bite on a distal tooth might produce an elevated bite force, this expectation is mitigated by the constraint that muscle force may be reduced during such a bite to prevent joint distraction. In theory, there is an alternative means of moving the muscle resultant into the triangle of support, namely, by reducing the anterior-most fibres of each muscle so as to move the muscle resultant posteriorly. This is difficult to model but, regardless, the effect on bite force is the same: reduced muscle forces necessarily imply reduced bite forces.

**Figure 5:**
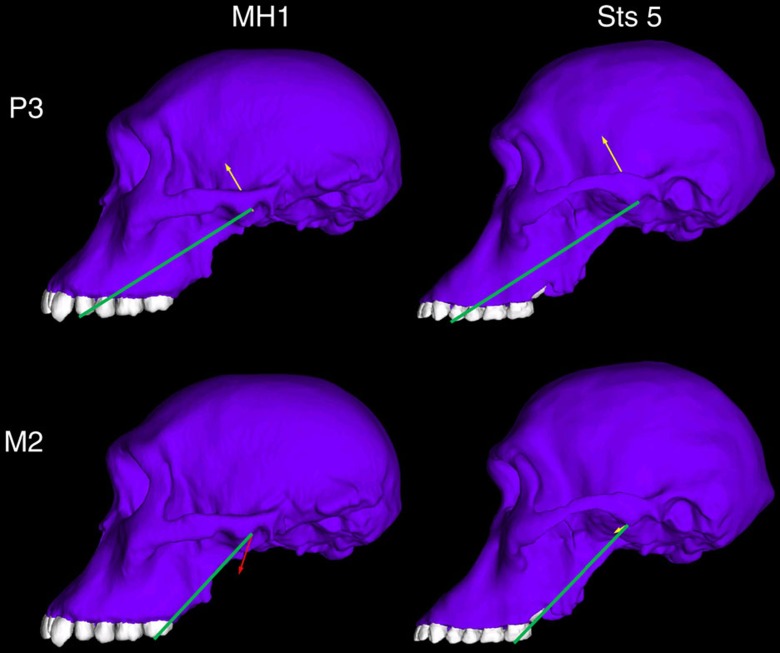
Orientation of the joint reaction force at the working (biting) side temporomandibular joint (TMJ) in models of (MH1) (*A. sediba*) and Sts 5 (*A. africanus*) during simulated left premolar (P^3^) and left molar (M^2^) biting. Arrows indicate direction of the reaction force. Yellow arrows indicate a compressive force, while red arrows indicate a distractive force, relative to the plane of the triangle of support (green line). Note that the zygomatic root (off of which the masseter muscle arises) is more mesially positioned relative to the tooth row in MH1 than Sts 5. Models are not shown to scale.

Taxa in which the molar teeth are positioned antero-posteriorly close to the TMJs (that is, as in a species with a retracted face) are especially at risk of having a midline muscle resultant fall outside of the triangle of support and this problem is exacerbated if those taxa also have masticatory muscles that are positioned anteriorly^28^. Thus, it is a paradox that a feeding apparatus configured precisely in a way that increases mechanical advantage (that is, by increasing the leverage of the chewing muscles while simultaneously reducing the load arm of the bite point) is also subject to a constraint requiring a reduction in balancing-side muscle force that limits bite force production. The molar teeth in *A. sediba* are positioned relatively close to the TMJs^13^, and the origin of the masseter muscle is positioned towards the mesial end (rather than the middle) of the molar row (Fig. 5). Thus, one might expect that *A. sediba* was at risk of experiencing distractive joint forces during molar biting.

In our FEA simulations, the TMJs on the working (biting) sides of both the Sts 5 and MH1 crania experience compressive reaction forces during premolar biting, as predicted by biomechanical models^27^,^28^. This indicates that the jaw adductor muscle resultant vector passed safely through the triangle of support. Sts 5 similarly exhibits a faintly compressive reaction force during molar biting, which is consistent with the hypothesis that anthropoid primates may be buffered against TMJ distraction as muscle activity levels vary dynamically during molar mastication^28^. However, the working-side TMJ of the MH1 cranium experiences a distractive reaction force during maximal molar biting (Fig. 5; Table 1). Only one of the six chimpanzee models experiences a distractive TMJ force^29^, and in that individual the absolute value of the distractive force was an order of magnitude less than that recorded in MH1. Mammals avoid distractive TMJ reaction forces by reducing the activity levels of the chewing muscles on the balancing (non-biting) side of the skull^27^,^28^. The exact pattern by which they do this is difficult to predict, but it was found that when the balancing-side muscle forces in MH1 were all equally reduced by nearly 30%, then the MH1 model produced a working-side joint reaction force during molar biting that was not distractive (Table 1), and that had an orientation similar to that of Sts 5. This reduction in muscle forces in MH1 resulted in a maximum bite force that was reduced by ∼15%. We expect that in life, both *A. africanus* and *A. sediba* would have exhibited further reductions in balancing-side muscle force to maintain a safety factor to avoid dislocating the working-side TMJ^28^, as has been observed in modern humans^30^. Indeed, the nearly 30% value falls comfortably within the dispersion of experimental values observed during biting on the distal-most molar in modern humans^30^, who can exhibit even a 50% reduction (note that in MH1, the second molar is its distal-most tooth owing to its subadult age). Because scaled chimpanzee muscle forces are only a coarse proxy for muscle forces in the model of MH1, it is possible that differences in muscle size and/or force ratios could impact the results for the TMJ reaction forces, and potentially the conclusions relating to constraints on feeding biomechanics in *A. sediba*. To examine this further, we ran the premolar and molar biting simulations in MH1 a third time, using the muscle forces of another closely related species, *Homo sapiens*. Using these forces, it was found that balancing-side muscle force reductions necessary to eliminate distraction at the working side were only half those needed when using chimpanzee forces (Supplementary Table 3). Nonetheless, it is clear that the potential for muscle recruitment is more limited in MH1 than in Sts 5, and that the feeding apparatus in MH1 is not well buffered against TMJ distraction during molar chewing. Although MH1 would likely have been able to produce bite forces high enough to fracture some hard foods, our simulation suggests that the cranium of this specimen is not optimized to produce high bite force on the molars. Moreover, while MH1 could have efficiently produced bite force on the premolars, the presence of small premolar teeth in the conspecific specimen MH2 argues against the likelihood that premolar loading of hard foods with a high bite force was a significant behavior in *A. sediba* because the size of a tooth limits its maximum strength^31^. Thus, if these specimens are broadly representative of the species, our results do not support a hypothesis that *A. sediba* was adapted to eat hard foods. Previous analyses of carbon isotopes and dental calculus reveal that MH1 may have had a varied diet consisting of the tissues (including bark) of a range of plants utilizing the C_3_ photosynthetic pathway^7^. Our findings are consistent with this hypothesis, but suggest that hard-object feeding may not have been a behaviour that drove craniofacial evolution in *A. sediba*.

## Discussion

Dental microwear analysis suggests that MH1 and MH2 may have eaten hard foods shortly before their deaths^7^, and that microwear in these specimens resembles that of *Paranthropus robustus*, for which it is claimed^1^ that hard foods were a critical dietary resource consumed during parts of the year. Our mechanical data do not dispute the possibility that *A. sediba* occasionally ate hard foods (notwithstanding disagreements about how to interpret microwear data^32^), but limitations on bite force production suggest that hard foods needing to be processed with high bite forces were not a selectively important component of the diet of this species. This interpretation is consistent with the presence of smaller (and, thus, weaker^31^) cheek teeth in *A. sediba* than in many other australopiths. Moreover, insofar as MH1 was especially vulnerable to TMJ distraction, one can infer that molar bite forces in *A. sediba* would have been habitually low to minimize the magnitude of the joint forces. This implies that the foods most important to its survival may not have been mechanically challenging to process in the oral cavity. Thus, the dietary reconstruction of *A. sediba* based on dental microwear^7^ could be correct, yet not fully elucidate the nature of dietary adaptations in this species.

Our results highlight the influence of functional constraints on reconstructions of adaptations in extinct organisms; it may be as informative to consider the ways in which a given morphology limits behaviour as it is to consider how it allows a behaviour. Those limitations critically affect our assessments of adaptive hypotheses. Such hypotheses can be tested only by examining all relevant sources of information, including not only direct evidence of behaviour but also functional anatomy.

The phylogenetic relationships of *A. sediba* are a topic of debate. It has been hypothesized that *A. sediba* lies near the ancestry of *Homo* or is otherwise a close phylogenetic relative of the *Homo* clade^13^, but it has also been asserted that *A. sediba* is instead a phyletic descendant of *A. africanus* lacking especially close affinities to *Homo*^14^. Under both scenarios, our study has similar implications for the origin of *Homo.* If *A. sediba* and *Homo* are closely related, and if specimens drawn from the base of the *Homo* radiation exhibit limitations on molar bite force production similar to that found in *A. sediba* (as would be likely in *Homo* specimens exhibiting facial retraction), then it is reasonable to infer that these shared biomechanical constraints (caused especially by a short horizontal distance between the molar teeth and the TMJs) and their attendant behavioural limitations characterize the ancestors of our genus. These ancestors were almost certainly descended from gracile australopiths, for example, refs 13, 33, 34, 35, 36, 37, 38, who had adaptations for feeding on mechanically resistant foods, for example, refs 8, 9, 10, 29, 39, 40. In this scenario, we hypothesize that environmental change in the late Pliocene and/or early Pleistocene^41^,^42^,^43^ disrupted the ecological niches of gracile australopiths necessitating changes in dietary ecology. One clade of descendants accentuated adaptations for eating mechanically resistant foods for example, ref. 29, and evolved into the robust australopiths of the genus *Paranthropus*. Another clade (including *A. sediba)* seemingly abandoned the ability to process such foods orally and one lineage within this clade evolved into *Homo.* Large brains evidently do not characterize all members of this clade (for example, MH1), so the onset of this evolutionary trajectory may have preceded the evolution of brain expansion that so markedly characterizes our genus.

Alternatively, if *A. sediba* and *Homo* lack a close phylogenetic relationship, then any biomechanical similarities shared by them must have evolved in parallel. This would be consistent with a hypothesis that some gracile australopith populations were experiencing selection favouring craniodental reduction, even as the robust australopiths were evolving along an opposite trajectory. Thus, even if *A. sediba* and *Homo* were descended from different gracile australopith ancestors, it is evident that one must understand the dietary selective pressures that influenced the ancestors that preceded the earliest members of *Homo* if one is to understand the origin of the genus.

## Methods

### Model construction

A completely closed (‘watertight') virtual reconstruction of the MH1 cranium suitable for FEA was constructed in Geomagic Studio 2012 (Research Triangle Park, NC, USA) three-dimensional (3D) surface editing software from a stereolithography-formatted surface mesh rendered from computed tomography (CT) scans of the original specimen. This surface reconstruction was used to generate a FEM. Although the individual represented by the MH1 cranium is juvenile, any additional growth is unlikely to have significantly altered its morphology (see below).

The MH1 cranium is missing most of its right parietal, much of the posterior aspect of the right zygomatic arch, and the occipital. Regions preserved on the contralateral side, including the parietal and zygomatic arch, were reflected to the missing side (Supplementary Fig. 1). Missing teeth, with the exception of the central incisors, were also reflected from the opposite side. Central incisors were incorporated into the model using a surface rendering of an isolated central incisor attributed to the same individual (Supplementary Fig. 2). The occipital was reconstructed by incorporating surfaces from our model of Sts 5 (*A. africanus*; Supplementary Fig. 3). This region is not expected to play a role in feeding biomechanics, so the incorporation of these parts should not affect our results in any meaningful way.

There is some distortion to the sphenoid region of the model where it was difficult to discern bone from the surrounding matrix. This also became difficult in the frontal bone, so the frontal sinus was ultimately modelled from a scaled version of the frontal sinus of Sts 5. Further, the unreconstructed cranium of MH1 preserves a minor displacement of the rostrum, with the face being slightly bent to the left. Some of this displacement was corrected by rotating the displaced parts to the right, such that the point between the central incisors was aligned with the midsagittal plane (Supplementary Fig. 2). There is still some minor distortion to the cranium; however, the current geometry of the reconstructed model is a first-order approximation suitable for the purposes of our analysis.

The surface model of the reconstructed cranium was further refined in Geomagic Studio. During this stage, small cracks, holes and other minor damage to the cranium were corrected through light sanding and smoothing procedures. Any additional geometric adjustments necessary for successful solid meshing of the model, including the correction of overlapping or intersecting triangles, and removal of surface polygons with unusually high aspect ratios were performed using the same procedure. Volumes representing the trabecular bone (as opposed to individual trabeculae) in the supraorbital region, zygomatic and midface surrounding the tooth roots were also generated using Geomagic Studio. These volumes were reconstructed using the MH1 CT data as a guide. However, the clarity of the MH1 CT scans was somewhat too poor to precisely determine cortical thickness in certain facial regions. The source of this problem is that mineral matrix has partially or completely filled the cavities within the MH1 cranium, including the spaces between trabeculae. When this happens, it is sometimes difficult to discern the boundary between cortical bone (which has been mineralized) and the volume representing cancellous bone. Therefore, data on craniofacial cortical bone thickness in chimpanzees and gorillas gathered during our analysis of extant ape bone material properties^26^ were also used to determine reasonable cortical bone thicknesses across the face. Cortical bone thickness in the MH1 FEM is approximate.

The completed surface mesh of the MH1 cranium was then imported into the 3Matic module of Mimics v 14.0 (Materialise, Ann Arbor, MI, USA) for volume meshing. The individual watertight volumes representing the cranial cortical bone, trabecular bone in the supraorbital and zygomatic regions, and trabecular bone in the maxilla were volume meshed using four-noded tetrahedral elements. The solid mesh of the cranium was then imported as a Nastran file into Strand7 (Strand7 Pty Ltd, Sydney, NSW) finite element software for assignment of material properties, muscle forces and all loading simulations. The mandible of MH1 was also reconstructed so that insertion points for the jaw adductors could be determined (see below). Using Geomagic Studio, the mandibular molars of the right hemi-mandible were aligned to the right maxillary molars, and the mandibular condyle was inserted into the glenoid fossa. The mandible was then reflected to the missing side (Supplementary Fig. 4).

Model creation for *A. africanus* followed a procedure similar to that described above. Our model of *A. africanus* is an updated composite of Sts 5 and Sts 52a described in a previous analysis^10^. This new model incorporates an altered spatial relationship of the dentition following a new macrowear-based reconstruction of the dental arches in Sts 52a and b^16^. Specifically, the teeth have been slightly repositioned to better account for distortion in Sts 52a, and the tooth roots have been more precisely aligned with the preserved apices of the alveoli of Sts 5. These subtle changes are, for the purposes of this study, biomechanically insignificant. The FEM of MH1 is available for download at www.biomesh.org.

### Material properties

The material properties of cranial cortical bone in our FEMs are averaged values from one chimpanzee and one gorilla specimen^26^ and were obtained using an ultrasonic technique^44^,^45^ at 14 homologous locations^46^ across the cranial vault and facial skeleton (Supplementary Fig. 5). Spatially heterogeneous isotropic material properties were spread throughout the cortical volume of the models in Strand7 software using a method^47^ analogous to the diffusion of heat through a highly conductive material. To achieve this, each FEM was assigned thermally conductive properties, allowing heat to diffuse smoothly throughout the model (with any effect of temperature on stress resistance during the loading analyses removed by assigning a thermal expansion of 0). Nodes at the 14 locations on each side of the cranium were seeded with temperatures that correspond to their respective elastic moduli (Supplementary Table 4), and the steady-state (non-transient) thermal problem was solved to determine the temperature distribution in the cranium (Supplementary Fig. 6). The elastic moduli of each cortical volume at various locations across the face were then set to vary with the changes in temperature during the loading analyses described below. Trabecular bone and tooth crowns were assigned homogeneous isotropic material properties, with moduli of 0.637 and 80 GPa, respectively, each with a Poisson's ration of 0.3, following our previous analyses^10^,^26^,^29^. Periodontal ligaments were not modelled because our prior research has shown that the presence or absence of the periodontal ligament does not have a major effect on global patterns of cranial bone strain in a primate model^48^.

### Muscle forces and loading conditions

Muscle forces were applied to both FEMs for the anterior temporalis, superficial masseter, deep masseter and medial pterygoid. Force magnitudes were estimated from the physiological cross-sectional area of each of these muscles in an adult female chimpanzee^10^, but were scaled to bone volume to the 2/3 power in each FEM to remove any size-related differences in strain pattern or magnitude^18^. This focuses our comparison on the functional consequences of differences in shape alone. Although the scaling of muscle forces controls for the confounding effects of size, this approach necessarily requires the assumption that the relative magnitudes of each of the muscles remains constant across species. There is no reliable way of assessing this assumption because it has been shown that bony muscle markings are an inaccurate indicator of muscle cross-sectional area^49^, which is in turn related to maximum force magnitude. Thus, to assess the sensitivity of the models to variation in relative muscle force magnitudes, analyses were rerun using scaled human muscle forces. Human muscle forces applied to the MH1 model were derived from data on muscle cross-sectional area^50^ and adjusted using formulae that correct for the effects of gape during fixation on sarcomere length^51^. Results were similar insofar as it was still found that bilaterally symmetrical muscle forces produced distractive TMJ reaction forces during molar bites, although less reduction in balancing-side muscle force was needed to eliminate this distraction (Supplementary Table 3).

To apply muscle forces to the FEMs, plate elements were ‘zipped' at their nodes to surface elements representing each muscle's origin. The scaled muscle forces were applied to the plate elements using a software package (Boneload) that uses an algorithm that applies both tangential and normal muscle tractions, accounting for the added torque produced when muscles wrap around curved bone surfaces^52^. These loads were directed towards their respective insertions on the mandible. Muscle insertion points were defined as the 3D area centroid of each muscle's insertion area using Area Centroids (www.BioMesh.org). To account for changes in muscle vector orientation during mouth opening, insertion points were determined with the mandible of each FEM slightly depressed and with the condyles translated onto the articular eminences^53^.

Each FEM was subjected to two primary loading experiments simulating premolar and molar biting. For both analyses, the working-side TMJ was constrained against translation in all directions, while the balancing-side TMJ was constrained in the vertical and anteroposterior directions. This creates an axis of rotation upon the application of muscle forces. During the premolar simulation, a node in the centre of the occlusal surface of the left upper third premolar (P^3^) was constrained in the vertical direction. The left upper second molar (M^2^) was similarly constrained for the molar biting simulation. These constraints induce deformation in the craniofacial skeleton and generate reaction forces at constrained nodes when loaded by muscle forces.

### Comparative sample of chimpanzees

FEMs were also constructed of a comparative sample of chimpanzees. Due to the time-consuming nature of FEA, this sample consisted of six individuals shown to differ markedly in morphology (thereby bracketing a large proportion of intraspecific morphological variation). Geometric morphometric methods were used to select these six specimens, and those methods are described fully elsewhere^26^. Briefly, as part of a previous study^54^, 709 cranial landmarks and semilandmarks were digitized from 3D surfaces derived from the CT scans of 21 adult chimpanzees, including 10 females, 9 males and 2 individuals of indeterminate sex. These specimens sample at least two chimpanzee subspecies (*Pan troglodytes schweinfurthii* and *Pan troglodytes verus*). The (semi)landmark configurations were converted to shape coordinates by generalized Procrustes analysis, and principal component analysis was then used to detect the main patterns of shape variation across our chimpanzee sample^26^. The specimens with the strongest positive and negative loadings along the first three principal components (PCs) were selected for FEA, with the caveat that, by chance, the specimens loading at the extreme ends of PC2 were associated with CT scans whose quality was too poor for the purposes of building a model for FEA (segmentation to reconstruct the full suite of internal and external geometry was not feasible, although scans were sufficient for gathering shape data). Thus, on PC2, we selected the specimens with the next most extreme loadings on both the positive and negative axes.

### Ontogenetic prediction and assessment of shape change in MH1

3D landmark data were analysed to assess the degree to which the MH1 facial morphology might have changed during the final stages of ontogeny. Landmarks were chosen to test the relative positions of the malar root, TMJ and dento-palatal morphology, which are key features in the FEM. These include interdental alveolar landmarks between all teeth from I^1^ to M^2^, the malar root origin, and the tip of the postglenoid process, and were collected in the manner described by McNulty^55^. A cross-sectional ontogenetic sample of 319 extant African apes and humans was used to represent possible developmental trajectories for *A. sediba* (cf. samples in McNulty^19^); polytypic species were represented by samples from only a single subspecies (*Gorilla gorilla gorilla* and *Pan troglodytes troglodytes*). All landmark configurations, including that of MH1, were superimposed using a generalized Procrustes analysis to project specimens into a common shape space^56^,^57^.

To contextualize the degree of developmental change subsequent to second molar occlusion, a principal component analysis was performed on mean configurations at different developmental stages. For each species, means were computed for specimens with (a) no permanent molars (complete deciduous dentition), (b) M^2^ in occlusion but M^3^ unerupted and (c) complete dental occlusion. Separate means were computed for adult males and females due to their divergent morphology^58^. Principal component ordination of these mean configurations summarizes the relevant shape differences between M^2^ occlusion and adults, relative to the longer developmental sequence during permanent molar eruption (Fig. 3a).

Developmental changes after M^2^ occlusion were also assessed visually by generating hypothetical adult morphologies of MH1 using species- and sex-specific developmental trajectories of extant African apes and humans. Developmental vectors were computed by subtracting the M^2^ sample means from the adult means. For each species × sex, these vectors represent the shape differences between the average of the M^2^ configuration and the average adult configuration. Each of these vectors was then added to the MH1 landmark configuration to generate a new set of landmarks representing the estimated adult morphology of MH1 according to the species × sex vector. A surface rendering of MH1 based on synchotron X-ray tomography data^59^ was warped into these hypothetical configurations using thin-plate spline interpolation computed in Landmark 3.0.0.6 (ref. 60). While all adult configurations were examined, the hypothetical adult morphology is represented here using the ‘morph' computed from the *Pan troglodytes* developmental vector (Fig. 3b).

### Collection of strain data

FEA provides information about multiple types of stress, strain and deformation at each element in a FEM, resulting in potentially millions of variates (although these variates are not statistically independent from each other). Colour maps, as in Fig. 2, provide a convenient means of summarizing large amounts of data, allowing qualitative assessments of strain magnitude and distribution. Quantitative data were extracted from a small number of locations on the crania (Fig. 1) corresponding to regions from which strain data have been collected *in vivo* during primate feeding experiments or that are found on morphological traits that are derived in australopiths^26^,^29^. Strain data from 10 of the regions from which *in vivo* data exist were then used to ‘normalize' the colour maps in Fig. 2 so as to provide information about the spatial distribution of high and low strain magnitudes independent of absolute strain magnitudes (strain from the zygomatic arches were excluded from this procedure because those data vary so extensively *in vivo* that they would have heavily biased the resulting relative strain maps^26^).

## Additional information

**How to cite this article:** Ledogar, J. A. *et al*. Mechanical evidence that *Australopithecus sediba* was limited in its ability to eat hard foods. *Nat. Commun.* 7:10596 doi: 10.1038/ncomms10596 (2016).

## Supplementary Material

Supplementary Information Supplementary Figures 1-6, Supplementary Tables 1-4 and Supplementary Reference

## Figures and Tables

**Table 1:** Force inputs and outputs.

	MH1 symmetrical* **muscle forces**	MH1 asymmetrical* **muscle forces**	**Sts 5 symmetrical muscle forces**
Model volume (mm^3^)	305,801	305,801	347,264
W anterior temporalis force†	527.04	527.04	573.66
B anterior temporalis force	527.04	374.20	573.66
W superficial masseter force	542.09	542.09	590.05
B superficial masseter force	542.09	384.89	590.05
W deep masseter force	80.62	80.62	87.76
B deep masseter force	80.62	57.24	87.76
W medial pterygoid force	179.13	179.13	194.98
B medial pterygoid force	179.13	127.18	194.98
Total applied muscle force	2,658	2,272	2,893^2^
P^3^ bite force	1,043	--	1,178
P^3^ mechanical advantage‡	0.39	--	0.41
M^2^ bite force	1,827	1,557	1,786
M^2^ mechanical advantage	0.69	0.69	0.62
W TMJ reaction force: P^3^ bite§	310.68	--	454.78
B TMJ reaction force: P^3^ bite	845.63	--	842.50
W TMJ reaction force: M^2^ bite	−154.81	1.69	48.44
B TMJ reaction force: M^2^ bite	624.78	399.26	685.73

B, balancing; TMJ, temporomandibular joint; W, working.

^*^MH1 was analysed twice using scaled chimpanzee muscle forces, once with bilaterally symmetrical muscle forces and once with asymmetrical muscle forces. This was necessary because the MH1 FEM exhibited an unrealistic distractive reaction force at the working (biting) side TMJ during molar biting with symmetrical muscle forces. To remove the distractive reaction force, it was necessary to reduce the balancing (non-biting) muscle forces by nearly 30% and rerun the simulation of molar biting. The analysis using asymmetrical forces is a more realistic simulation of molar biting in this specimen.

^†^All forces (muscle, bite and reaction) are in newtons (N).

^‡^Mechanical advantages for each bite point were calculated as the ratio of bite force output to the total muscle force input.

^§^Positive TMJ reaction forces are compressive, while negative TMJ reaction forces are distractive (tensile).
