# Supplementary material for: Mechanical evidence that Australopithecus sediba was limited in its ability to eat hard foods
Source: Nat Commun. 2016 Feb 8;7:10596. doi: 10.1038/ncomms10596 (PMC4748115; doi:10.1038/ncomms10596)
Supplement: Supplementary Information — Supplementary Figures 1-6, Supplementary Tables 1-4 and Supplementary Reference [file ncomms10596-s1.pdf]

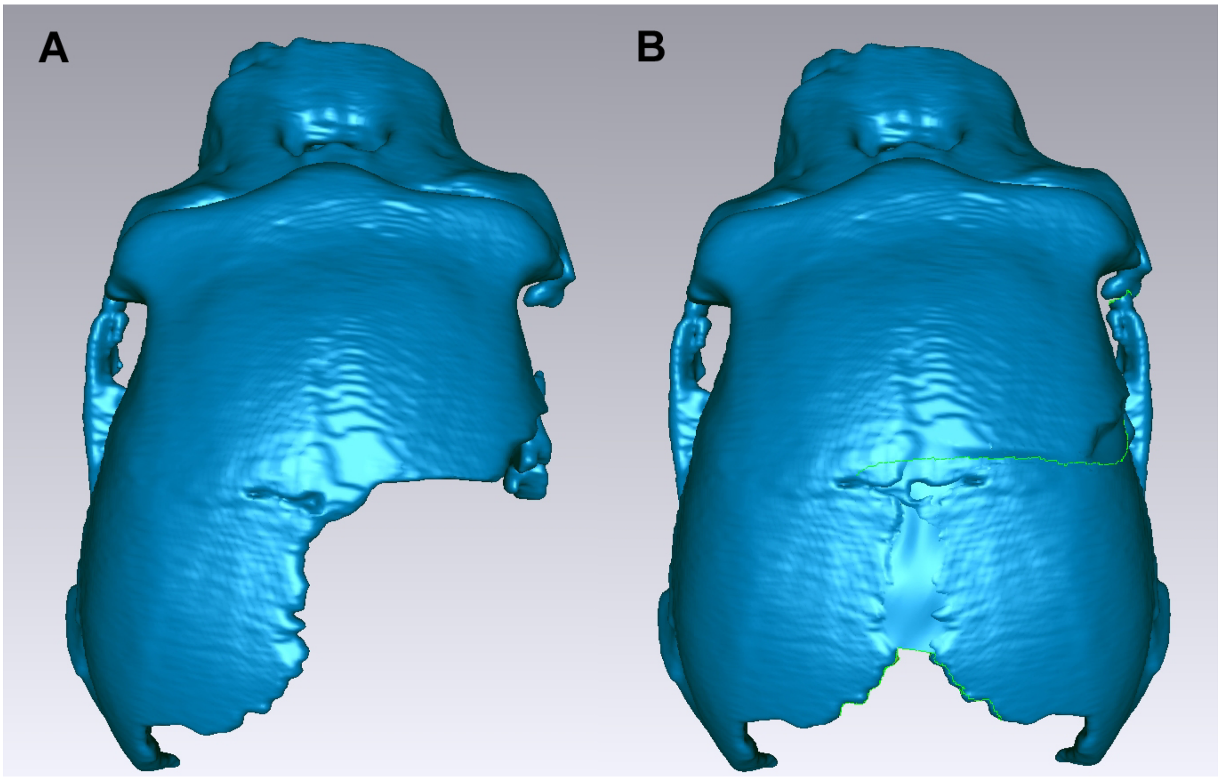

Supplementary Fig. 1. (A) Superior view of the original (unreconstructed) surface of the MH1 cranium, and a **(B)** superior view of the original MH1 surface with the left parietal and zygomatic arch reflected to the right side.

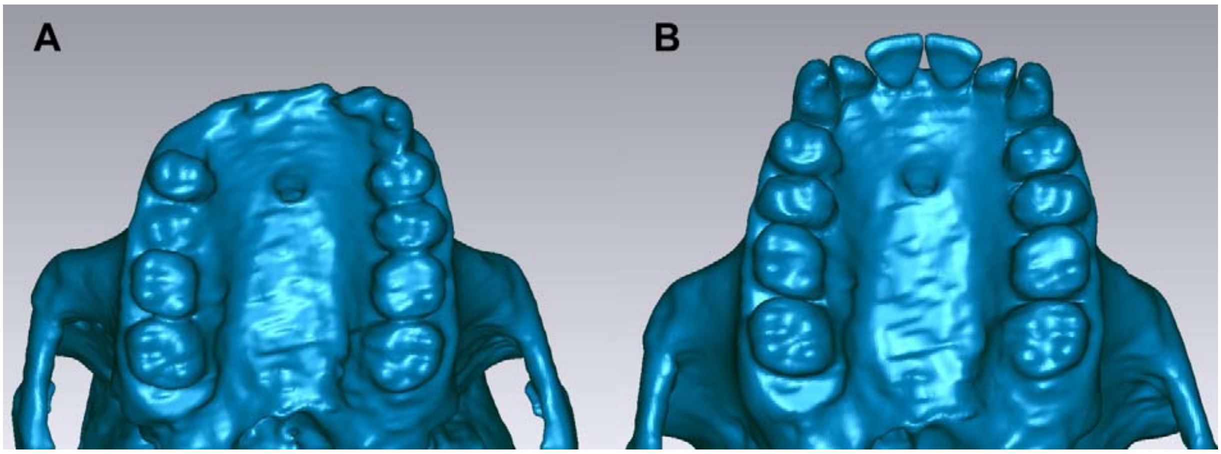

Supplementary Fig. 2. Inferior view of the **(A)** original (unreconstructed) palate and dental arcade of MH1, and the **(B)** reconstructed MH1 dental arcade. These screenshots also capture before and after correction of the rotational displacement of the lower face.

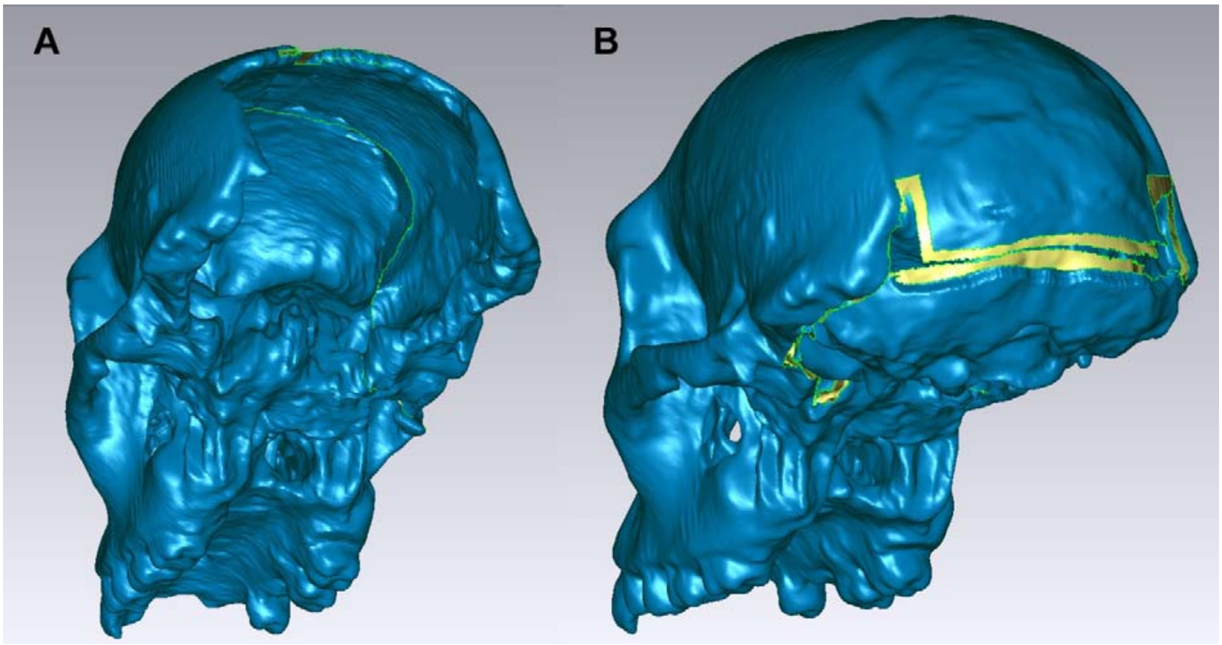

Supplementary Fig. 3. Oblique views of the posterior aspect of the MH1 surface showing the missing occipital region **(A)**, and the reconstruction of the posteroinferior portions of the cranium in progress using scaled occipital elements from Sts 5 **(B)**.

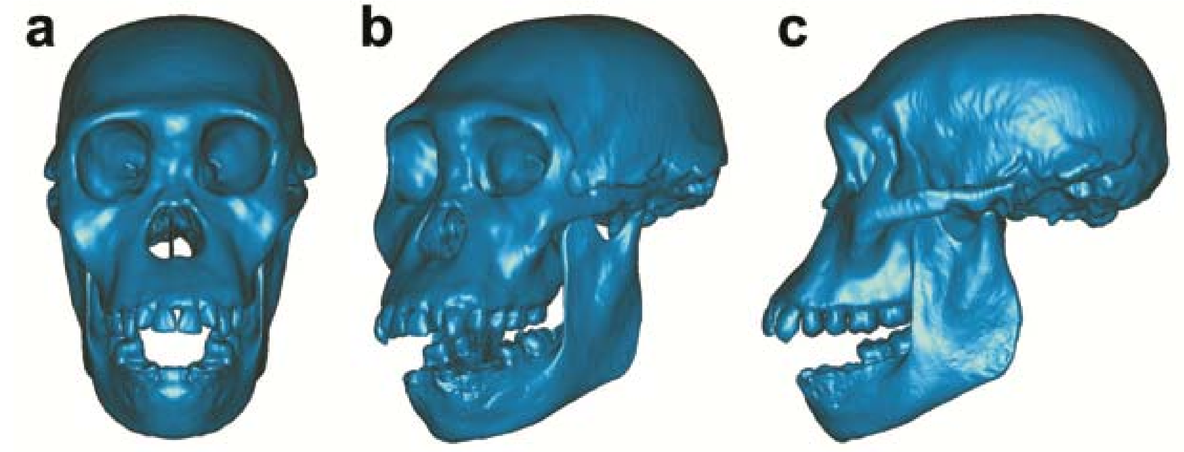

Supplementary Fig. 4. (A) Anterior, **(B)** oblique, and **(C)** lateral views of the heuristically reconstructed surface of the *Australopithecus sediba* holotype cranium and mandible (MH1) used to create a finite element model. Note that the mandible was only used to determine jaw adductor insertion sites.

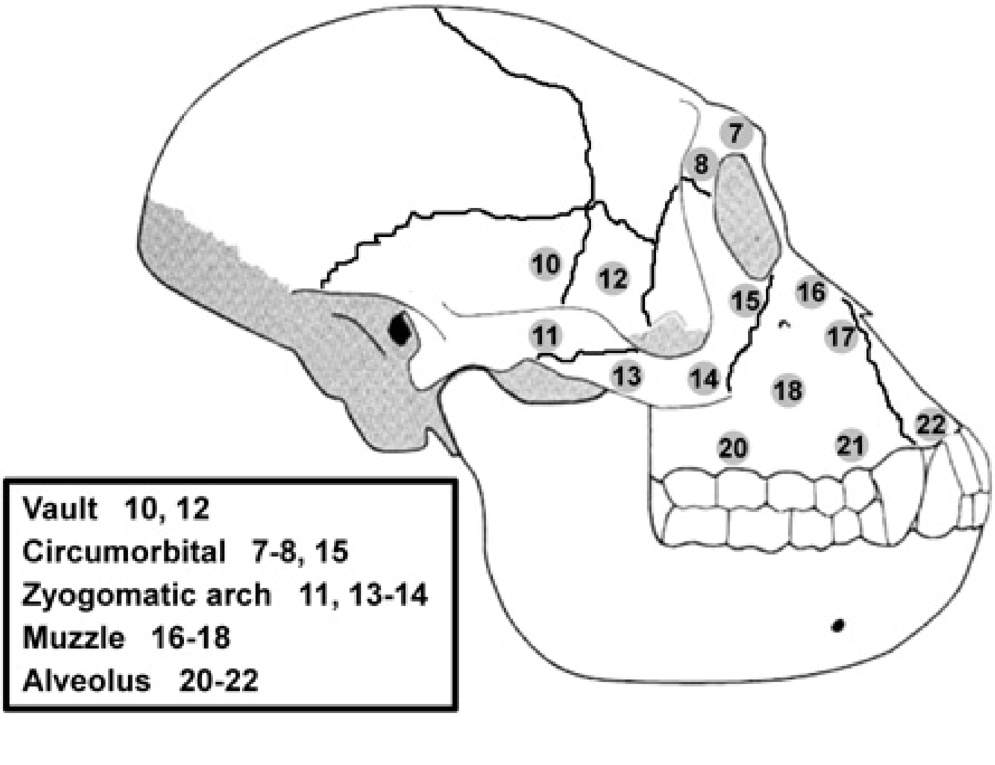

Supplementary Fig. 5. The 14 locations from which material properties of chimpanzee and gorilla craniofacial cortical bone were collected. Note that the numbers for each location are not consecutive and only correspond to those assigned during data collection.

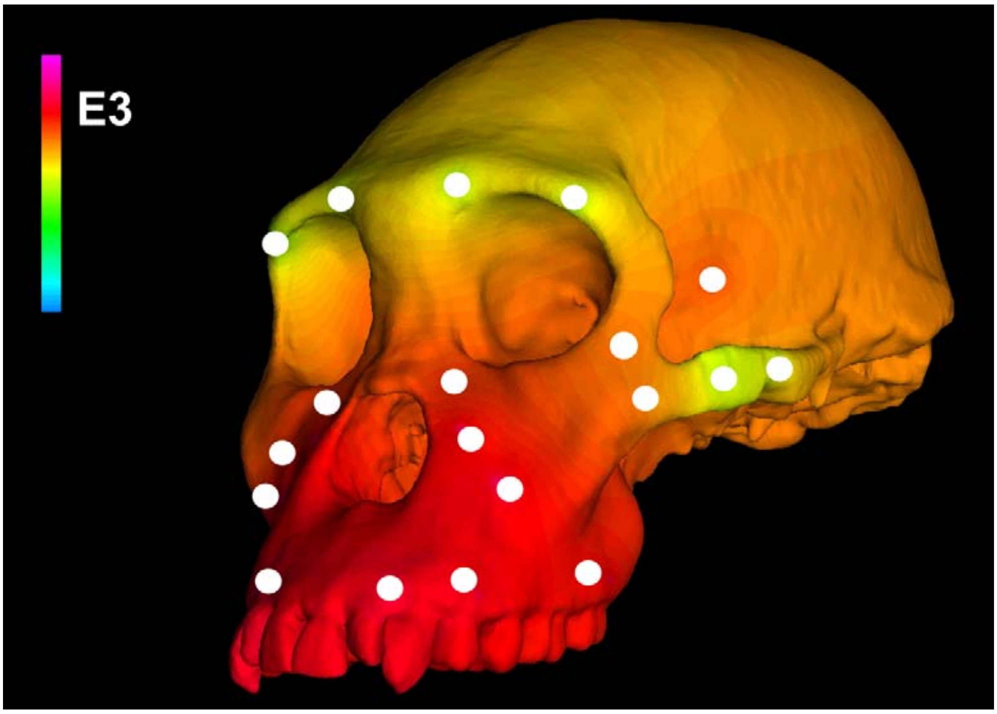

Supplementary Fig. 6. Thermal diffusion of elastic modulus through the cranium of MH1. “Warmer” colors depict regions of high stiffness (elastic modulus), while “cooler” colors depict regions of lower stiffness.

Supplementary Table 1. Strain and strain energy density (SED) results from simulated premolar (P^3) bites*.

Location	Specimen	Max Prin (με)	Min Prin (με)	Mode	Max shear (με)	von Mises (με)	SED (J/mm³)
1. Dorsal interorbital	MH1	78	-38	2.05	116	106	0.04
	Sts 5 / 52	186	-93	2	279	243	0.29
	Chimp range	118-216	-48 to -125	1.31-3.17	171-288	160-271	0.10-0.34
2. W Dorsal orbital	MH1	67	-42	1.6	109	98	0.03
	Sts 5 / 52	119	-99	1.2	218	189	0.13
	Chimp range	16-138	-21 to -88	0.61-2.27	37-199	32-189	0.00-0.14
3. B Dorsal orbital	MH1	82	-52	1.58	134	124	0.05
	Sts 5 / 52	102	-38	2.68	140	136	0.07
	Chimp range	106-197	-48 to -101	1.89-3.10	153-291	146-268	0.08-0. 28
4. W postorbital bar	MH1	234	-264	0.89	498	431	0.66
	Sts 5 / 52	581	-412	1.41	993	872	2.76
	Chimp range	150-455	-168 to -481	0.89-1.42	318-935	276-810	0.27-2.33
5. B postorbital bar	MH1	385	-194	1.98	579	527	1.08
	Sts 5 / 52	910	-402	2.26	1312	1217	5.92
	Chimp range	370-769	-184 to -479	1.41-2.43	553-1248	504-1115	0.99-4.56
6. W Zygo arch	MH1	219	-288	0.76	507	445	0.71
	Sts 5 / 52	856	-1135	0.75	1991	1784	11.26
	Chimp range	337-1710	-492 to -3390	0.36-1.63	859-5006	757-4791	2.04-83.65
7. B Zygo arch	MH1	369	-349	1.06	718	622	1.39
	Sts 5 / 52	1044	-2255	0.46	3299	3164	36.73
	Chimp range	417-1994	-179 to -2683	0.39-2.99	713-3958	686-3549	1.71-53.64
8. W Zygo root	MH1	197	-560	0.35	757	712	2.25
	Sts 5 / 52	449	-1000	0.45	1449	1300	7.5
	Chimp range	245-537	-514 to -979	0.34-0.76	905-1339	788-1202	2.26-7.50
9. B Zygo root	MH1	163	-211	0.77	374	330	0.38
	Sts 5 / 52	120	-441	0.27	561	520	1.52
	Chimp range	101-347	-275 to -539	0.35-1.15	387-693	350-644	0.64-3.03
10. W Infraorbital	MH1	382	-319	1.2	701	608	1.33
	Sts 5 / 52	472	-216	2.19	688	636	1.6
	Chimp range	385-575	-211 to -479	1.06-1.82	762-1054	560-916	1.12-2.99
11. B Infraorbital	MH1	246	-157	1.57	403	360	0.47
	Sts 5 / 52	480	-182	2.64	662	635	1.62
	Chimp range	199-477	-146 to -351	1.21-2.05	346-828	304-722	0.33-1.92
12. W Nasal margin	MH1	218	-600	0.36	818	803	2.52
	Sts 5 / 52	407	-1213	0.34	1620	1600	10.29
	Chimp range	207-356	-463 to -891	0.35-0.66	679-1242	625-1183	1.54-5.60
13. W Zygo body	MH1	387	-250	1.55	637	564	1.17

	Sts 5 / 52	454	-229	1.98	683	632	1.5
	Chimp range	536-994	-375 to -593	1.27-1.83	911-1576	793-1424	2.38-7.45
14. B Zygo body	MH1	314	-145	2.17	459	430	0.71
	Sts 5 / 52	585	-293	2	878	783	2.57
	Chimp range	343-1506	-198 to -739	1.27-3.15	520-2245	502-2048	0.90-16.48

* Chimp ranges from Smith et al.[1]. W = working, B = balancing.

Supplementary Table 2. Strain and strain energy density (SED) results from simulated molar (M^2) bites*.

Location	Specimen	Max Prin (με)	Min Prin (με)	Mode	Max shear (με)	von Mises (με)	SED (J/mm^3)
1. Dorsal interorbital	MH1	75	-32	2.34	107	102	0.04
	Sts 5 / 52	172	-81	2.12	253	222	0.24
	Chimp range	108-198	-45 to -76	1. 46-3.10	153-270	145-250	0.08-0.28
2. W Dorsal orbital	MH1	59	-24	2.46	83	80	0.02
	Sts 5 / 52	118	-102	1.16	220	191	0.13
	Chimp range	5-81	-22 to -91	0.23-2.18	27-138	26-120	0.00-0.07
3. B Dorsal orbital	MH1	93	-55	1.69	148	139	0.07
	Sts 5 / 52	102	-39	2.62	141	136	0.07
	Chimp range	110-209	-46 to -111	1.80-3.08	156-310	150-284	0.09-0.31
4. W postorbital bar	MH1	161	-241	0.67	402	356	0.46
	Sts 5 / 52	357	-381	0.94	738	640	1.45
	Chimp range	93-365	-150 to -431	0.62-1.21	243-796	216-692	0.17-1.70
5. B postorbital bar	MH1	413	-187	2.21	600	556	1.22
	Sts 5 / 52	935	-381	2.45	1316	1234	6.2
	Chimp range	413-817	-169 to -492	1.43-2.54	536-1309	492-1175	0.97-5.08
6. W Zygo arch	MH1	175	-265	0.66	440	392	0.56
	Sts 5 / 52	875	-1115	0.78	1990	1776	11.19
	Chimp range	293-1629	-445 to -3416	0.37-1.62	737-5039	662-4832	1.57-84.99
7. B Zygo arch	MH1	353	-333	1.06	686	595	1.27
	Sts 5 / 52	1064	-2253	0.47	3317	3171	36.81
	Chimp range	405-1955	-172 to -2675	0.39-3.00	686-3950	666-3542	1.61-53.31
8. W Zygo root	MH1	259	-749	0.35	1008	971	3.95
	Sts 5 / 52	718	-1721	0.42	2439	2188	22.51
	Chimp range	350-570	-523 to -1061	0.38-0.74	910-1475	795-1803	2.30-8.41
9. B Zygo root	MH1	158	-230	0.69	388	346	0.43
	Sts 5 / 52	127	-494	0.26	621	576	1.93
	Chimp range	108-351	-288 to -607	0.28-1.14	415-843	375-747	0.72-1.95
10. W Infraorbital	MH1	333	-218	1.53	551	485	0.88
	Sts 5 / 52	360	-195	1.85	555	504	0.96
	Chimp range	438-578	-227 to -498	1.16-2.06	718-1076	632-935	1.51-3.11
11. B Infraorbital	MH1	227	-134	1.69	361	326	0.39
	Sts 5 / 52	445	-141	3.16	586	574	1.39
	Chimp range	191-448	-135 to -307	1.21-2.21	325-754	287-660	0.30-1.63
12. W Nasal margin	MH1	69	-75	0.92	144	125	0.06
	Sts 5 / 52	136	-245	0.56	381	352	0.45
	Chimp range	111-254	-119 to -457	0.49-1.19	244-681	211-625	0.17-1.51
13. W Zygo body	MH1	381	-233	1.64	614	548	1.11

	Sts 5 / 52	460	-231	1.99	691	638	1.54
	Chimp range	566-993	-408 to -572	1.34-1.77	974-1697	847-1497	2.71-8.07
14. B Zygo body	MH1	294	-128	2.3	422	399	0.61
	Sts 5 / 52	576	-301	1.91	877	775	2.54
	Chimp range	329-1468	-171 to -727	1.40-3.13	500-2195	477-1996	0.81-15.71

* Chimp ranges from Smith et al.[1]. W = working, B = balancing.

Supplementary Table 3: Force inputs and outputs during simulated bites using scaled human muscle forces.

	MH1 with human symmetrical† muscle forces	MH1 with human asymmetrical† muscle forces
**W Anterior temporalis force**	121.70	121.70
**B Anterior temporalis force***	121.70	103.44
**W Superficial masseter force**	99.65	99.65
**B Superficial masseter force**	99.65	84.70
**W Deep masseter force**	50.50	50.50
**B Deep masseter force**	50.50	42.93
**W Medial pterygoid force**	102.96	102.96
**B Medial pterygoid force**	102.96	87.52
**Total applied muscle force**	750	693
**P^3 bite force**	272	--
**P^3 mechanical advantage‡**	0.36	--
**M^2 bite force**	477	441
**M^2 mechanical advantage**	0.64	0.64
**W TMJ reaction force: P^3 bite§**	106.19	--
**B TMJ reaction force: P^3 bite**	251.23	--
**W TMJ reaction force: M^2 bite**	-18.43	0.49
**B TMJ reaction force: M^2 bite**	189.55	157.59

* All forces (muscle, bite, reaction) are in newtons (N). W = working, B = balancing.

† As in the analyses that employed scaled chimpanzee muscle forces, MH1 was analyzed twice with the human muscle forces applied, once with bilaterally symmetrical muscle forces and once with asymmetrical muscle forces. This was necessary because the MH1 FEM again exhibited a distractive reaction force at the working (biting) side TMJ during molar biting with symmetrical muscle forces. In order to remove the distractive reaction force, it was necessary to reduce the balancing (non-biting) muscle forces and re-run the simulation of molar biting. However, the necessary balancing side reduction necessary to eliminate distraction when applying scaled human muscle forces was 15%, roughly half of what was required when using scaled chimpanzee muscle forces.

‡ Mechanical advantages for each bite point were calculated as the ratio of bite force output to the total muscle force input.

§ Positive TMJ reaction forces are compressive, while negative TMJ reaction forces are distractive (tensile).

Supplementary Table 4: Material properties thermally diffused through finite element models*.

Location	Elastic modulus (GPa)	Poisson's ratio (*v*)
Alveolus - 20	18.36	0.318
Alveolus - 21	18.36	0.318
Alveolus - 22	19.40	0.318
Circumorbital - 7	12.63	0.318
Circumorbital - 8	10.58	0.318
Circumorbital - 15	17.05	0.318
Muzzle - 16	17.81	0.318
Muzzle - 17	18.72	0.318
Muzzle - 18	19.74	0.318
Vault - 10	18.43	0.318
Vault - 12	19.05	0.318
Zygomatic arch - 11	12.66	0.318
Zygomatic arch - 13	13.98	0.318
Zyogomatic arch - 14	16.45	0.318

* Data on elastic modulus are averaged values from one chimpanzee and one gorilla[1]. Locations numbered as in Supplementary Fig. 5.

Supplementary Reference

1. Smith, A. L. *et al.* Biomechanical implications of intraspecific shape variation in chimpanzee crania: moving towards an integration of geometric morphometrics and finite element analysis. *Anat. Rec.* **298,** 122-144 (2015).
